# Deep learning‐based cortical thickness maps for diagnosis of neurodegenerative diseases: a rater study

**DOI:** 10.1002/dad2.70458

**Published:** 2026-08-25

**Authors:** Evamaria O. Riedel, Fabian Bongratz, Benedikt Zott, Cornelius Berberich, Paul Eichinger, Igor Yakushev, Timo Grimmer, Christian Wachinger, Dennis M. Hedderich

**Affiliations:** ^1^ Department of Neuroradiology School of Medicine and Health, TUM Klinikum Rechts der Isar, Technical University of Munich Munich Germany; ^2^ Department of Radiology School of Medicine and Health, TUM Klinikum Rechts der Isar, Technical University of Munich Munich Germany; ^3^ Munich Center for Machine Learning Munich Germany; ^4^ Department of Nuclear Medicine School of Medicine and Health, TUM Klinikum Rechts der Isar, Technical University of Munich Munich Germany; ^5^ Department of Neurology School of Medicine and Health, TUM Klinikum Rechts der Isar, Technical University of Munich Munich Germany

**Keywords:** Alzheimer's disease, brain, deep learning, dementia, frontotemporal lobar degeneration, magnetic resonance imaging

## Abstract

**INTRODUCTION:**

Cortical atrophy patterns on magnetic resonance imaging (MRI) are essential for diagnosing neurodegenerative diseases (NDs), but remain challenging to assess visually. Deep learning enables quantitative evaluation of cortical thickness (CTh) and z‐score maps.

**METHODS:**

3T three‐dimensional T1 MRI from 40 ND patients (Alzheimer's dementia, posterior cortical atrophy, behavioral variant frontotemporal dementia, semantic variant primary progressive aphasia) and 10 controls were retrospectively analyzed. Cortical surfaces were extracted with FreeSurfer and registered to fsaverage, and z‐score maps were generated using stochastic cortical self‐reconstruction (SCSR). Three neuroradiologists rated CTh or z‐score maps, each with or without 3D T1 for ND presence and differential diagnosis. Conventional 3D T1 served as baseline reading condition.

**RESULTS:**

Diagnostic accuracy (Acc) was quantitatively highest for z‐score maps with 3D T1 (Acc = 0.98) for the detection of ND, though it did not reach statistical significance. However, diagnostic confidence improved for z‐score maps versus baseline 3D T1 (adjusted *p =* 0.017 for Rater 2). Inter‐rater agreement improved from *κ =* 0.525 (baseline 3D T1) to *κ =* 0.792 (z‐score with 3D T1).

**DISCUSSION:**

SCSR‐generated z‐score maps show promise for diagnostic evaluation of ND.

## Highlights

- Evaluation of deep learning‐generated z‐score maps for diagnosis of NDs.
- Z‐score maps may improve diagnostic accuracy and the exclusion of NDs.
- Z‐score maps increase clinician confidence and inter‐rater agreement.

## 1 BACKGROUND

Neurodegenerative diseases (NDs), such as Alzheimer's disease (AD) and rarer forms like frontotemporal dementia (FTD) and posterior cortical atrophy (PCA), are among the leading causes of global morbidity and mortality,^1^, ^2^, ^3^ with rising prevalence in aging populations and improved diagnostic capabilities.^2^, ^4^ A decrease in cortical thickness (CTh) is a biomarker for neurodegeneration, possibly due to neuronal loss.^5^, ^6^ Disease‐specific patterns of cortical thinning^7^, ^8^, ^9^, ^10^ may aid differential diagnosis.

Magnetic resonance imaging (MRI) offers detailed insights into brain structure and atrophy patterns^11^; however, subtle cortical atrophy – especially in early or atypical presentations – can be difficult to assess visually on MRI. The intricate folding of the cortex and the fact that cortical thinning often occurs at subvoxel levels (typically < 1 mm)^12^ make visual detection difficult and highly dependent on the rater's expertise, introducing variability and limiting diagnostic consistency. Advanced postprocessing tools such as FreeSurfer,^12^ CAT12,^13^ and Vox2Cortex^14^ reconstruct cortical surfaces and measure CTh at high resolution (per vertex).

Reference charts, widely used in clinical medicine to assess typical versus atypical development (e.g., growth curves), have also been introduced for CTh.^15^ These charts provide age‐related norms, but they are defined on brain parcels rather than vertices and do not easily extend to new data due to imaging site effects.^15^ Normative models aim to quantify how much an individual deviates from a healthy population but remain relatively scarce in the context of CTh. Their interpretability is often limited by measurement noise and site and scanner effects.

To address these limitations, the deep learning framework of stochastic cortical self‐reconstruction (SCSR)^16^ does not rely on a normative model. Instead, it generates individualized, vertex‐level z‐score maps to highlight atypical CTh by constructing individualized healthy references from MRI‐derived CTh data by randomly corrupting cortical regions and reconstructing them using information from the remaining cortical structures. Through repeated self‐reconstruction, SCSR generates a stochastic reference cortex that implicitly accounts for potential confounding factors, thereby detecting subtle deviations from typical cortical patterns. In summary, individual vertex‐level deviat maps can be generated reliably and quickly, potentially providing biomarker information for differential diagnosis.

**RESEARCH IN CONTEXT**

1. **Systematic review**: We utilized PubMed to identify previous studies that assessed the clinical use of cortical thickness (CTh) analysis for the differential diagnosis of NDs. CTh analysis has been shown to be sensitive to disease‐related changes and useful in differential diagnosis. Z‐score maps have previously not been assessed in a clinical rater study for this indication.
2. **Interpretation**: Our findings demonstrate that the visualization of CTh deviations in individualized, deep learning‐generated cortical z‐score maps may improve the detection of neurodegenerative patterns on MRI and provide an intuitive and interpretable approach to support clinical decision‐making.
3. **Future directions**: Prospective evaluation in larger cohorts and longitudinal follow‐up are needed. Integration into routine clinical workflows is essential.

We conducted a clinical rater study to evaluate the added value of CTh as well as z‐score maps compared to standard visual reading of MRI in patients with distinct neurodegenerative disorders. Specifically, we investigated whether these maps can enhance diagnostic accuracy, confidence, and consistency across different ND types.

## 2 METHODS

### 2.1 Ethics statement

This non‐interventional, retrospective study was approved by the local ethics committee (2025‐411‐S‐CB) and conducted in accordance with the ethical standards of the 1964 Declaration of Helsinki and its subsequent updates.^17^

### 2.2 Consent statement

Individual informed consent was not required for this non‐interventional, retrospective study using anonymized data, as approved by the local ethics committee.

### 2.3 Patient selection

We retrospectively searched our local Picture Archiving and Communication System for patients imaged as part of routine clinical practice between 2011 and 2018 and consecutively enrolled 12 subjects per group (AD, behavioral variant [bvFTD], semantic variant primary progressive aphasia [svPPA], PCA, healthy controls [HCs]). Inclusion criteria were as follows: age > 18 years; diagnosis of AD, bvFTD, svPPA, or PCA according to international criteria^5^, ^18^, ^19^, ^20^; clinical and positron emission tomography (PET)‐MRI confirmation; representative regional atrophy on MRI as assessed by an attending neuroradiologist; and availability of 1 mm isotropic three‐dimensional (3D) T1. All participants underwent simultaneous 18F‐fluorodeoxyglucose (FDG) PET‐MRI. Amyloid‐ or tau‐targeted PET was not utilized. We required concordance between clinical symptoms, MRI atrophy, and FDG‐PET hypometabolism; cases with discordant findings or major structural abnormalities – including mass lesions, hydrocephalus, evidence of prior neurosurgery, or large cortical infarcts – were excluded. Only subjects with mild, age‐related vascular changes (Fazekas scale score ≤ 1) were eligible for inclusion.

**FIGURE 1:**
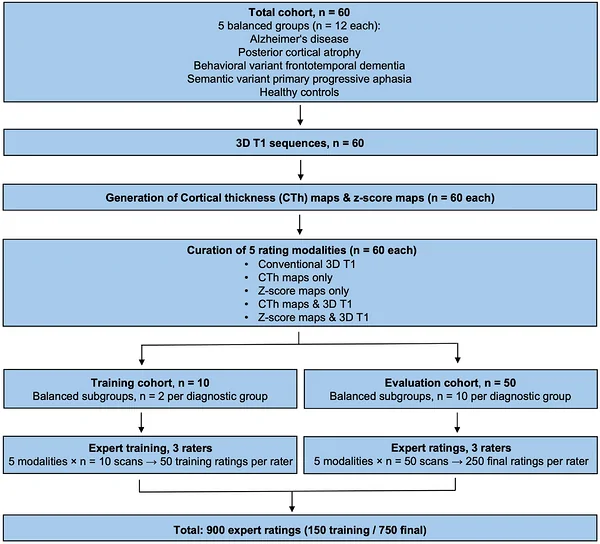
Workflow. 1‐mm 3D T1w sequences from a total of 60 patients with different forms of dementia (AD, bvFTD, svPPA, PCA) and healthy controls were exported from our PACS, and cortical thickness (CTh) as well as cortical z‐score maps were generated. After familiarizing themselves with the maps using a training cohort of *n =* 10, three expert raters rated *n =* 50 participants in five scenarios: conventional 3D T1 images only, CTh maps only, z‐score maps only, CTh maps combined with 3D T1, and z‐score maps combined with 3D T1.

HCs were >18 years old, free of dementia or major neurological conditions (e.g., stroke, tumor), had unremarkable MRI reports, and had a 1 mm 3D T1 (Figure 1). To ensure balanced comparisons and avoid class imbalance, we deliberately limited enrollment to 12 subjects per group.

All T1‐weighted scans successfully completed the FreeSurfer pipeline and underwent visual quality control to inspect the accuracy of the white and pial surface boundaries. No scans were excluded due to motion, incomplete coverage, or segmentation errors.

### 2.4 MRI scans and data export

MRI was performed by healthcare professionals during routine clinical practice on a 3T PET/MRI hybrid scanner (Siemens Biograph). Patients underwent unenhanced imaging, including at least a 3D T1 magnetization prepared rapid gradient echo with 1 mm isotropic voxels (repetition time [TR] 2300 ms, echo time [TE] 2.98 ms, inversion time [TI] 900 ms, slice thickness 1 mm, interslice gap 0 mm, acquisition matrix (freq × phase) 256 × 240, in‐plane resolution 1.0 mm × 1.0 mm, field of view = 256 × 240 mm, flip angle = 9°). Images were pseudonymized, exported as DICOM, and converted to NIfTI using dcm2nii.^21^ Age and sex at scan time were recorded as demographic variables with the scans.

### 2.5 Generation of cortical thickness maps

T1‐weighted MRI scans were processed using FreeSurfer version 7.2^12^ (recon‐all) to estimate CTh from reconstructed cortical surfaces. Spatial correspondences across subjects' cortices had to be established to work at the vertex level. We adhered to FreeSurfer's procedure, which includes inflation (mris_inflate), mapping to the sphere (mris_sphere), spherical registration (mris_register), and resampling (mri_surf2surf). The registration was performed using the fsaverage template,^22^ with the region labeled as “unknown” in the DKT atlas (medial wall) and corpus callosum ignored. The resampling target was a fifth‐order icosphere, providing 10,242 CTh measurements per hemisphere.

### 2.6 Generation of z‐score maps

Individualized z‐score maps were generated using the SCSR framework,^16^ implementing the standard multilayer perceptron (MLP)‐based flat autoencoder variant. The network features three latent layers (1024 dimensions each), with input and output dimensions corresponding to the resolution of the fifth‐order icosphere (*p =* 10,242 vertices per hemisphere). Batch normalization layers with Swift activation function were positioned between the layers, and a dropout layer with a rate of 0.5 was applied at the second layer. The underlying SCSR model was previously trained on a large‐scale, population‐based dataset of healthy individuals from the UK Biobank (UKB) imaging study. This cohort was randomly split into training and validation sets, yielding a final training sample of *n =* 25,338 healthy subjects (mean age: 64.0 ± 7.5 years, age range: 45 to 82 years; 47.3% male). Crucially, there was no subject overlap between this independent UKB dataset and our clinical evaluation cohort. During model training, an iterative vertex masking and reconstruction strategy was used: A random sampling rate of 20% was selected to mask vertices, and non‐sampled vertices were set to zero (corresponding to the mean after standard scaling). Optimization was performed using AdamW (learning rate = 0.001, weight decay = 0.005) over 200 epochs, and the checkpoint demonstrating the lowest mean squared error (MSE) reconstruction loss on the non‐sampled regions of the UKB validation set was selected.

For our evaluation cohort, vertex‐wise z‐scores were computed by comparing the actual thickness to the reconstructed reference, which was defined as the 95th centile from 100 reconstruction iterations. To standardize these deviation maps, the individual reconstruction residuals (the difference between actual and predicted CTh) were normalized vertex‐wise using the mean (μ) and standard deviation (σ) of the reconstruction residuals previously established across the independent healthy UKB validation dataset:

$$z_{v}=\frac{\left(\mathrm{Residua}l_{\mathrm{patient},v}-\mu_{\mathrm{validation},v}\right)}{\sigma_{\mathrm{validation},v}},$$

where *v* denotes the vertex index. This statistical normalization yields localized, surface‐based z‐score maps that reflect individual structural deviations from an expected healthy baseline.

### 2.7 Visualization of surfaces

Cortical surfaces were rendered using PyVista^23^ (version 0.35.2) in Python version 3.7.0. on inflated fsaverage templates^22^ to facilitate inter‐subject comparison and improve the visibility of cortical features. Using the inflated surface reduces the obscuration of values that would otherwise be hidden within the sulcal folds of the native, folded cortical geometry. All visualizations were generated with consistent color scales and orientation.

### 2.8 Expert ratings

**FIGURE 2:**
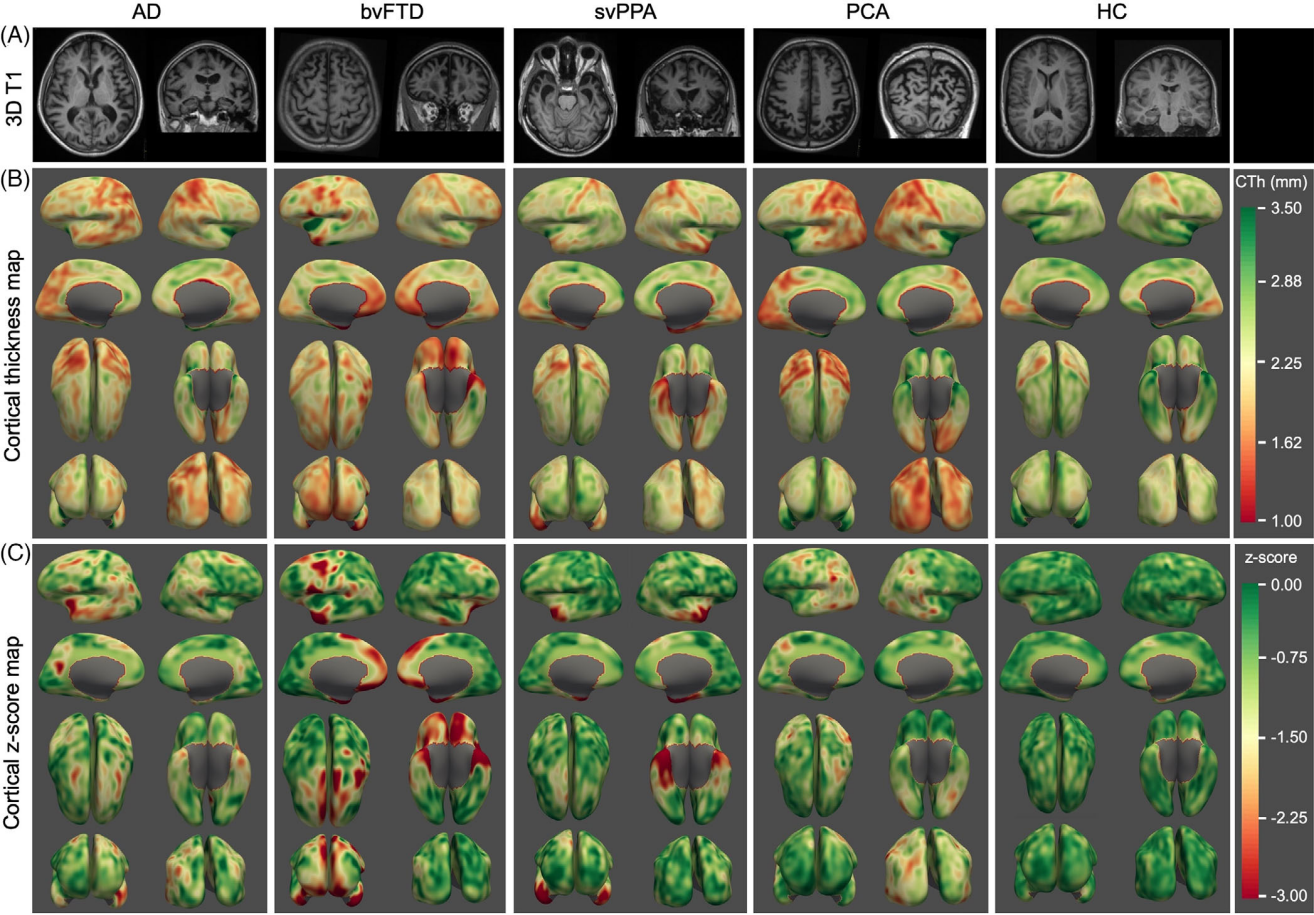
Cortical thickness and z‐score maps. Examples of conventional 3D T1 reading condition (A), corresponding cortical thickness maps (B), and z‐score deviation maps (C) for Alzheimer's disease (AD), behavioral variant frontotemporal dementia (bvFTD), semantic variant primary progressive aphasia (svPPA), posterior cortical atrophy (PCA), and for healthy controls (HCs). Gray regions refer to the region labeled as “unknown” in the DKT atlas (medial wall) and corpus callosum.

Three independent raters evaluated the cases: a neuroradiology resident with 2 years of subspecialty experience in neuroradiology (Rater 1); a board‐certified radiologist with 5 years of clinical experience, including 3 years of subspecialty training in neuroradiology (Rater 2); and an attending neuroradiologist with 11 years of clinical experience, including 5 years of specialized training in neuroradiology (Rater 3). After training on 10 baseline cases (two per subgroup), each rater assessed 50 cases (10 per subgroup). Ratings included (i) presence of ND and (ii) disease subtype (AD, bvFTD, svPPA, PCA), along with diagnostic confidence scored on a 3‐point scale (1 low, 2 medium, 3 high). Five conditions were tested: (1) 3D T1 alone, (2) CTh maps, (3) z‐score maps, (4) CTh maps with 3D T1, and (5) z‐score maps with 3D T1. To manage workload, conditions 4 and 5 were rated after a wash‐out period of 2 to 8 weeks after conditions 1 to 3. Cases were independently randomized within each of the five scenarios. Raters were blinded to diagnosis and group distribution; age and sex were provided. Maps were displayed in 2D Portable Document Format files showing lateral, medial, superior, and inferior views (Figure 2).

### 2.9 Statistical analysis

We calculated accuracy, sensitivity, specificity, positive predictive value (PPV), and negative predictive value (NPV) with Wilson confidence intervals. Binary diagnostic outcomes were analyzed using pairwise McNemar tests with Benjamini‐Hochberg (BH) correction to control for multiple comparisons. Two‐sided pairwise Wilcoxon signed‐rank tests were applied for the ordinal confidence ratings, with BH correction for multiple testing. Inter‐rater reliability among the three raters was assessed using Fleiss's kappa. The 95% confidence intervals for Fleiss's kappa were calculated based on the asymptotic standard error. For detecting ND versus HCs, all patients were included; subtype assessment was restricted to cases classified with ND. All analyses were performed in R^24^ version 4.4.2 using the packages dplyr version 1.1.4, tidyr version 1.3.1, DescTools version 0.99.60, and irr version 0.84.1. Plots were generated using the ggplot2 package version 3.5.2.

## 3 RESULTS

### 3.1 Patient population

The dataset comprised a training set of 10 participants (two per disease group and two controls) to familiarize the raters with the imaging maps and an evaluation set of 50 participants (10 per disease group and 10 controls). In the training set mean age was 58.4 (range 36 to 69); 50% of the participants were male. In the evaluation set mean age was 63.6 (range 43 to 82), with 38% male. AD: 46 to 82 years (mean* =* 64.4), 60% male; bvFTD: 43 to 79 years (mean* =* 59.9), 20% male; svPPA: 56 to 78 years (mean* =* 65.7), 50% male; PCA: 48 to 77 years (mean* =* 65.5), 20% male; and HC: 54 to 72 years (mean* =* 62.5), 40% male.

### 3.2 Cortical thickness and z‐score maps

CTh and z‐score maps were successfully generated for all participants and visualized on inflated cortical surfaces (Figure 2). The CTh color scale ranged from 1.0 to 3.5 mm. Across all disease subgroups and HCs, the postcentral gyrus appears thinner – consistent with its neuroanatomical structure. SCSR‐derived z‐score maps (standardized deviations from individualized healthy references) ranged from −3.00 to 0, with lower values indicating greater thinning.

### 3.3 Detection of NDs

**FIGURE 3:**
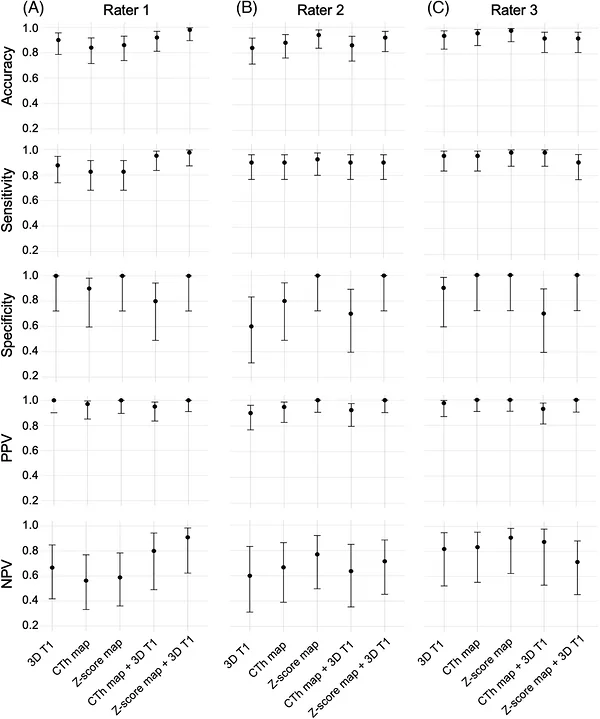
Diagnostic performance metrics. Means of point estimates with 95% confidence intervals calculated using the Wilson method for the detection of neurodegenerative diseases versus healthy controls for (A) Rater 1, (B) Rater 2, and (C) Rater 3 for the five different rating scenarios.

For the detection of NDs versus controls, confusion matrices are shown in Table S1. Accuracies were generally high. For Rater 1 the highest accuracy was observed with z‐score maps + 3D T1 (Acc = 0.98), followed by CTh maps + 3D T1 (Acc = 0.92; Table 1A, Figure 3A). For Raters 2 and 3 (Tables 1B,C, Figures 3B,C), the highest accuracy was observed for z‐score maps (Acc = 0.94 and 0.98) and for z‐score maps + 3D T1 (Acc = 0.92) and CTh maps (Acc = 0.96). There were no significant differences compared to the conventional 3D T1 reading condition using pairwise McNemar tests (Table S2). For all three raters, specificity and PPV reached 1.0 when using z‐score maps with and without 3D T1, reflecting perfect precision and reliability in positive classifications under these conditions. For Rater 1, combining maps with 3D T1 improved accuracy, sensitivity, and NPV, whereas Raters 2 and 3 did not benefit from the combined scenarios.

**TABLE 1:** Performance metrics (means of point estimates with 95% confidence intervals calculated using the Wilson method) for differentiation of neurodegenerative diseases versus healthy controls for (A) Rater 1, (B) Rater 2, and (C) Rater 3 for the five different rating scenarios.

Scenario	3D T1	CTh map	Z‐score map	CTh map + 3D T1	Z‐score map + 3D T1
**A. Rater 1**					
ACC	**0.900** (0.782 to 0.967)	**0.840** (0.708 to 0.928)	**0.860** (0.733 to 0.941)	**0.920** (0.808 to 0.977)	**0.980** (0.894 to 0.999)
Sens	**0.875** (0.732 to 0.958)	**0.825** (0.672 to 0.926)	**0.825** (0.672 to 0.927)	**0.950** (0.831 to 0.994)	**0.975** (0.868 to 0.999)
Spec	**1.000** (0.692 to 1.000)	**0.900** (0.555 to 0.997)	**1.000** (0.692 to 1.000)	**0.800** (0.444 to 0.975)	**1.000** (0.692 to 1.000)
PPV	**1.000** (0.900 to 1.000)	**0.971** (0.847 to 0.999)	**1.000** (0.894 to 1.000)	**0.950** (0.831 to 0.994)	**1.000** (0.910 to1.000)
NPV	**0.667** (0.384 to 0.882)	**0.563** (0.299 to 0.802)	**0.588** (0.329 to 0.816)	**0.800** (0.444 to 0.975)	**0.909** (0.587 to 0.998)
**B. Rater 2**					
ACC	**0.840** (0.709 to 0.928)	**0.880** (0.757 to 0.955)	**0.940** (0.835 to 0.987)	**0.860** (0.733 to 0.942)	**0.920** (0.808 to 0.977)
Sens	**0.900** (0.763 to 0.972)	**0.900** (0.763 to 0.972)	**0.925** (0.796 to 0.984)	**0.900** (0.763 to 0.972)	**0.900** (0.763 to 0.972)
Spec	**0.600** (0.262 to 0.878)	**0.800** (0.444 to 0.975)	**1.000** (0.692 to 1.000)	**0.700** (0.348 to 0.933)	**1.000** (0.692 to 1.000)
PPV	**0.900** (0.763 to 0.972)	**0.947** (0.823 to 0.994)	**1.000** (0.905 to 1.000)	**0.923** (0.791 to 0.984)	**1.000** (0.903 to 1.000)
NPV	**0.600** (0.262 to 0.878)	**0.667** (0.349 to 0.901)	**0.769** (0.462 to 0.950)	**0.636** (0.308 to 0.891)	**0.714** (0.419 to 0.916)
**C. Rater 3**					
ACC	**0.940** (0.838, 0.979)	**0.960** (0.865, 0.989)	**0.980** (0.895, 0.996)	**0.920** (0.812, 0.968)	**0.920** (0.812, 0.968)
Sens	**0.950** (0.835, 0.986)	**0.950** (0.835, 0.986)	**0.975** (0.871, 0.996)	**0.975** (0.871, 0.996)	**0.900** (0.769, 0.960)
Spec	**0.900** (0.596, 0.982)	**1.000** (0.722, 1.000)	**1.000** (0.722, 1.000)	**0.700** (0.397, 0.892)	**1.000** (0.722, 1.000)
PPV	**0.974** (0.868, 0.995)	**1.000** (0.908, 1.000)	**1.000** (0.910, 1.000)	**0.929** (0.810, 0.975)	**1.000** (0.904, 1.000)
NPV	**0.818** (0.523, 0.949)	**0.833** (0.552, 0.953)	**0.909** (0.623, 0.984)	**0.875** (0.529, 0.978)	**0.714** (0.454, 0.883)

### 3.4 Differentiation of ND subtypes

For disease subtype differentiation, we computed classification metrics per class with the corresponding confusion matrix shown in Table S3. For AD, accuracy was highest for z‐score maps (Acc = 0.84) for Rater 1. For Rater 2 it was highest for z‐score maps + 3D T1 (Acc = 0.83) and increased substantially from 3D T1 (Acc = 0.53) over z‐score maps (Acc = 0.73). Rater 3 showed the highest accuracy for z‐score maps with and without 3D T1 (Acc = 0.85 and 0.83; Table S4). For PCA, Rater 1 achieved the highest accuracy using z‐score maps (Acc = 0.94), while Rater 2 again performed best with the combination of z‐score maps and 3D T1 (Acc = 0.83); Rater 3 had the highest accuracy for CTh maps (Acc = 0.92; Table S5). For bvFTD, Raters 1and 2 achieved the highest accuracies for CTh maps (Acc = 0.88 and 0.97), followed by CTh maps + 3D T1 (Acc = 0.87 and 0.94). Rater 3 had the highest accuracy for z‐score maps (Acc = 0.92; Table S6). For svPPA, the highest accuracy was observed for CTh maps + 3D T1 for Rater 1 (Acc = 0.95) and for CTh maps with and without 3D T1 for Rater 2 (Acc = 0.94). For Rater 3, z‐score maps with and without 3D T1 yielded the highest accuracies (Acc = 0.94 to 0.95) (Table S7). There were no significant differences in pairwise comparisons against the conventional 3D T1 reading condition. Altogether, across disease subgroups, the highest accuracies for AD were achieved with z‐score maps alone or combined with 3D T1. Raters 1 and 2 had highest accuracies for PCA for z‐score maps with or without 3D T1 and for bvFTD and svPPA subtypes for CTh maps with or without 3D T1. Rater 3 showed an inverse pattern with the highest accuracies for PCA for CTh maps and for bvFTD and svPPA for z‐score maps.

### 3.5 Diagnostic confidence

**FIGURE 4:**
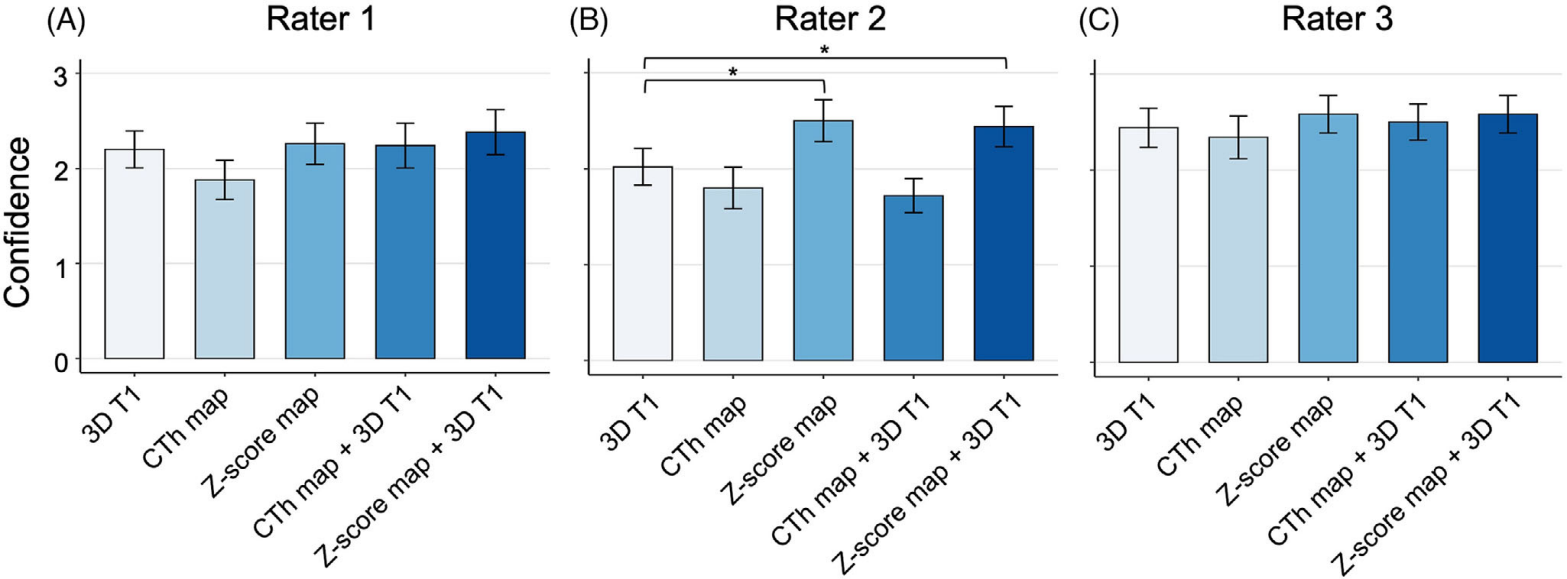
Confidence for detection of any neurodegenerative disease versus healthy controls. 1 = low, 2 = medium, 3 = high. Mean ± SEM for (A) Rater 1, (B) Rater 2 and (C) Rater 3; *p_adj ≤ 0.05.

Confidence ratings were compared for (i) the detection of any ND versus HCs and (ii) multiclass differentiation of ND subtypes. For (i), Rater 2 reported significantly higher confidence when using z‐score maps and z‐score maps + 3D T1, compared to baseline 3D T1 (*p*_adj_ = 0.017) (Figure 4, Table S8). For (ii), there were no significant differences over baseline 3D T1, Table S9.

### 3.6 Inter‐rater reliability

Fleiss's κ was calculated for (i) the detection of any ND, (ii) multiclass differentiation of ND subtypes, and (iii) per‐class differentiation of individual disease subtypes. For (i), inter‐rater agreement in increased from moderate using baseline 3D T1 (*κ =* 0.525) to substantial for z‐score maps + 3D T1 (*κ =* 0.792) (Table 2A). For (ii), we observed fair agreement for baseline 3D T1, moderate agreement for CTh maps and z‐score maps + 3D T1, and substantial agreement for CTh and z‐score maps (Table 2B). For (iii), the highest inter‐rater agreement was observed for AD and PCA using CTh maps (κ = 0.50 and 0.86, respectively) and for bvFTD and svPPA using z‐score maps (*κ =* 0.90 and 0.95, respectively). Generally, inter‐rater agreement was lowest for AD (*κ =* 0.17 to 0.50). The largest improvement was seen for svPPA for z‐score maps (*κ =* 0.95) versus baseline 3D T1 (*κ =* 0.21). Interestingly, across all four disease entities, inter‐rater agreement was lower for the combined scenarios with 3D T1 than for the scenarios with CTh or z‐score maps alone (Table S10).

**TABLE 2:** Inter‐rater agreement (Fleiss's κ) with 95% confidence intervals calculated using the asymptotic standard error method for (A) detection of any neurodegenerative disease versus healthy controls and (B) multiclass differentiation of neurodegenerative disease subtypes for the five different rating scenarios.

Fleiss's κ	3D T1	CTh map	Z‐score map	CTh map + 3D T1	Z‐score map + 3D T1
**A. Detection of any ND**	**0.525** (0.365, 0.685)	**0.625** (0.465, 0.785)	**0.698** (0.538, 0.858)	**0.615** (0.455, 0.775)	**0.792** (0.632, 0.952)
**B. Differentiation of NDs**	**0.323** (0.204, 0.442)	**0.707** (0.587, 0.826)	**0.785** (0.662, 0.909)	**0.466** (0.357, 0.576)	**0.478** (0.364, 0.593)

### 3.7 Qualitative expert feedback

All three raters considered z‐score maps as the most intuitive and effective tool, particularly for rapid separation of healthy and diseased cases. The combination of z‐score maps with 3D T1 was perceived to be especially beneficial. CTh maps were noted to be most informative for svPPA‐typical features.

## 4 DISCUSSION

This study examined the diagnostic utility of cortical maps from conventional CTh analysis (CTh maps) and the SCSR deep learning framework (z‐score maps) in MRI‐based assessment of NDs. Our results indicate that particularly z‐score maps, alone or combined with 3D T1‐weighted imaging, may modestly enhance diagnostic accuracy, confidence, and inter‐rater reliability.

CTh declines with age^25^ and shows disease‐specific atrophy patterns,^7^, ^20^, ^26^, ^27^ and previous work demonstrated its clinical utility for differentiating NDs.^25^, ^26^, ^28^ Approaches to better visualizing cortical thinning, such as normative brain volume reports and CTh analysis, are sensitive to disease‐related changes and useful in differential diagnosis,^29^, ^30^, ^31^ yet their integration into routine diagnostics remains limited. In our study, CTh maps added no value for differentiating ND from HCs. One rater benefited from combining CTh maps with 3D T1, whereas the other two showed stable or slightly reduced performance. While map‐only conditions were methodologically necessary to isolate the independent diagnostic signal, clinical application will likely center on combined use with conventional 3D T1 imaging.

The SCSR framework introduces a novel approach to generating individualized z‐score maps without relying on normative cohorts. Unlike models such as brain charts or other normative models, which require samples from the new hospital dataset for adaptation or calibration, SCSR operates without the need for such data. Traditional normative models (such as GAM, GAMLSS, or Bayesian Linear Regression) typically predict expected CTh at a macro‐regional or parcel level using demographic covariates like age and sex as inputs. Furthermore, while many traditional frameworks primarily focus on predicting absolute thickness values, SCSR goes a step further by utilizing validation residuals to output standardized, vertex‐wise z‐scores. This transforms raw anatomical measurements into statistical deviation metrics, allowing clinicians to immediately identify pathological atrophy without manually accounting for inter‐individual baseline variations. Shifting the paradigm from demographic to spatial prediction, SCSR operates directly at a high‐resolution vertex‐level (*p =* 10,242 vertices per hemisphere) and leverages iterative masking to estimate the healthy reference from the intact spatial correlations of the patient's own cortex. Consequently, it circumvents the need for site‐specific calibration or target‐dataset harmonization, providing a truly automated, site‐agnostic “out‐of‐the‐box” clinical tool. While z‐scores for volumetric data have shown promise,^32^ evaluations for CTh are scarce. Our findings suggest that SCSR‐based z‐score maps may moderatly enhance diagnostic accuracy with high precision in positive classifications within this cohort. For one rater, combining z‐score maps with 3D T1 substantially increased NPV, aiding exclusion of neurodegenerative disorders in this cohort. The rapid differentiation between healthy and diseased individuals using z‐score maps has potential clinical relevance, particularly when a preliminary diagnostic hypothesis already exists.

Direct comparisons with literature results are limited but conceptually align with studies using normative brain reports. Evidence for their benefit for human raters remains sparse. Pemberton et al. showed quantitative reports of regional brain volumes improved accuracy by >5%.^33^ Similarly, normative brain volume reports improved identification of ND for one rater (69% vs 88% correctly identified cases).^31^ Zaki et al. reported accuracies of 0.83 to 0.90 using two different normative brain report software tools,^34^ and Hedderich et al. found 0.74 to 0.83 without and 0.77 to 0.81 with reports for automated normative volumetry.^35^ Vernooij et al. noted a decreased accuracy for AD and FTD with quantitative reports alone but improved AD accuracy when combined with visual information.^36^ Rudolph et al. showed AUC gains (0.80 vs 0.93) for AD with artificial intelligence support.^37^ Like our findings, other studies reported no improvement in diagnostic accuracy for FTD.^33^, ^36^, ^37^ Importantly, baseline diagnostic accuracy for conventional 3D T1 in our study, exceeded values reported in comparable work.^31^, ^33^, ^35^ This high baseline likely created a statistical ceiling effect, restricting the scope for further improvement and suggesting that the observed gains represent a conservative lower bound for the potential value of CTh and z‐score maps.

Diagnostic confidence increased significantly for one rater when using z‐score maps alone and combined with 3D T1 in distinguishing ND from HCs. This indicates that z‐score maps may reduce diagnostic ambiguity and reinforce clinician certainty, particularly in early or unclear cases. Comparable studies using normative brain reports found no significant improvement in confidence,^33^, ^34^ though one study using a web‐based volumetry tool (volBrain) reported increased confidence for one of three raters.^35^

Higher inter‐rater reliability further supports the utility of z‐score maps. Agreement improved from moderate with baseline 3D T1 (0.53) to near perfect when z‐score maps were included (0.79), suggesting these maps can help standardize interpretation. Interestingly, the highest agreement occurred for AD and PCA with CTh maps and for bvFTD and svPPA with z‐score maps. Comparable studies using normative brain reports show mixed results: Hedderich et al. reported a similar increase (κ 0.56 to 0.85),^31^ while Vernooij et al. observed little change (0.69 visual, 0.62 normative, 0.67 combined).^36^ Zaki et al. found agreement ranging from 0.73 to 0.82 across two volumetry tools.^34^ For volBrain, reliability varied between raters.^35^ Pemberton et al. reported modest improvement, with Cronbach's alpha rising from 0.89 to 0.93.^33^ Overall, quantitative reports can enhance consistency, but their impact depends on disease context, report type, and rater experience.

Cortical z‐score maps generated through SCSR thus represent a novel advancement, offering a visually accessible, clinically adaptable tool for identifying structural deviations. Their individualized nature may capture subtle changes before they appear on conventional imaging or CTh mapping, particularly when conventional imaging is inconclusive. Inspired by stereotactic surface projection (SSP) used in FDG‐PET imaging, which compares individual scans to a normative database to highlight hypometabolism,^38^ SCSR can be viewed as a step toward establishing a MRI‐based counterpart to SSP maps, enabling individualized deviation mapping and potentially enhancing the interpretability of structural imaging.

The study has several limitations. Baseline diagnostic accuracy was high, exceeding values reported in comparable studies. While this demonstrates that SCSR provides stable utility even compared to a very high baseline, it also means that the added value of CTh deviation mapping may differ for general radiologists, residents, or non‐expert clinicians. The influence of prior training or familiarity with cortical maps remains uncertain and warrants further investigation. We intentionally restricted the clinical information provided to the raters to age and sex alone. While this design isolated the diagnostic utility of the imaging maps and eliminated confounding clinical bias, it represents a limitation as it deviates from real‐world clinical practice, where clinicians heavily rely on patient symptoms, history, and prior imaging. Additionally, this study is characterized by a single‐center, single‐scanner design. Future multicenter studies involving diverse scanner vendors and protocols are required to confirm the generalizability of the rater performances observed here. We acknowledge that our strict inclusion criteria resulted in a cohort of highly representative, congruent cases. While this was a deliberate and necessary methodological choice to establish baseline validation and evaluate our expert raters' performance, it may not fully capture the heterogeneity of routine clinical practice. Limitations further include the relatively small sample size and the consecutive enrollment strategy resulting in an artificial balancing across the five diagnostic groups. While the balanced design was deliberately chosen to ensure equal training and evaluation conditions across all conditions without class bias, it does not reflect the natural clinical prevalence of these neurodegenerative disorders. Consequently, this work should be considered a proof‐of‐concept study, and future research with larger, naturalistic clinical cohorts will be required to confirm the generalizability of our findings.

Future research should validate these approaches in larger, more heterogeneous cohorts with overlapping or ambiguous pathologies, assess early detection and longitudinal monitoring, and compare with normative models. Integration into routine workflows and exploration of specific visual features or rater biases underlying discrepancies between agreement and accuracy are also needed.

## 5 CONCLUSION

We assessed two approaches to visualizing cortical atrophy patterns in a retrospective rater study. SCSR‐derived cortical z‐score maps show potential to improve the visual evaluation of NDs via deviation mapping. Although the observed improvements in diagnostic accuracy metrics, confidence, and inter‐rater reliability were modest in this sample, this work provides a promising proof‐of‐concept foundation. Larger, more heterogeneous clinical studies are needed to clarify how cortical z‐score maps can be integrated into routine diagnostic workflows.

## CONFLICT OF INTEREST STATEMENT

The authors declare no conflicts of interest. Author disclosures are available in the Supporting Information.

## Supporting information




**Supporting Information**:

**Supporting Information**:

## Data Availability

Non‐imaging data are available from the corresponding author upon reasonable request.
