# Supplementary material for: Deep learning‐based cortical thickness maps for diagnosis of neurodegenerative diseases: a rater study
Source: Alzheimers Dement (Amst). 2026 Aug 25;18(3):e70458. doi: 10.1002/dad2.70458 (PMC13507028; doi:10.1002/dad2.70458)
Supplement: Supplementary file 2 [file DAD2-18-e70458-s002.docx]

Supplementary Material for

**Deep learning-based cortical thickness maps for diagnosis of neurodegenerative diseases: a rater study**

Evamaria O. Riedel, Fabian Bongratz, Benedikt Zott, Cornelius Berberich, Paul Eichinger, Igor Yakushev, Timo Grimmer, Christian Wachinger*, Dennis M. Hedderich*

^*^ These authors contributed equally to this work.

| Scenario | | 3D T1 | | CTh map | | z-score map | | CTh map  + 3D T1 | | z-score map  + 3D T1 | |
| --- | --- | --- | --- | --- | --- | --- | --- | --- | --- | --- | --- |
| A) Rater 1 | | **Predicted** | | | | | | | | | |
|  |  | **HC** | **ND** | **HC** | **ND** | **HC** | **ND** | **HC** | **ND** | **HC** | **ND** |
| Actual | **HC** | 10 | 0 | 9 | 1 | 10 | 0 | 8 | 2 | 10 | 0 |
|  | **ND** | 5 | 35 | 7 | 33 | 7 | 33 | 2 | 38 | 1 | 39 |
| B) Rater 2 | | **Predicted** | | | | | | | | | |
|  |  | **HC** | **ND** | **HC** | **ND** | **HC** | **ND** | **HC** | **ND** | **HC** | **ND** |
| Actual | **HC** | 6 | 4 | 8 | 2 | 10 | 0 | 7 | 3 | 10 | 0 |
|  | **ND** | 4 | 36 | 4 | 36 | 3 | 37 | 4 | 36 | 4 | 36 |
| C) Rater 3 | | **Predicted** | | | | | | | | | |
|  |  | **HC** | **ND** | **HC** | **ND** | **HC** | **ND** | **HC** | **ND** | **HC** | **ND** |
| Actual | **HC** | 9 | 1 | 10 | 0 | 10 | 0 | 7 | 3 | 10 | 0 |
|  | **ND** | 2 | 38 | 2 | 38 | 1 | 39 | 1 | 39 | 4 | 36 |

**Supplementary Table 1:** Confusion matrix for detection of neurodegenerative diseases (ND) versus healthy controls (HC) for (**A**) Rater 1, (**B**) Rater 2 and **(C)** Rater 3 for the five different rating scenarios.

|  | 3D T1 vs CTh | 3D T1 vs z-score | 3D T1 vs CTh map + 3D T1 | 3D T1 vs z-score map + 3D T1 |
| --- | --- | --- | --- | --- |
| Rater 1 | *p* = 0.450, *p*_adj_ = 1.000 | *p* = 0.683, *p*_adj_ = 1.000 | *p* = 1.000, *p*_adj_ = 1.000 | *p* = 0.134, *p*_adj_ = 0.802 |
| Rater 2 | *p* = 0.752, *p*_adj_ = 1.000 | *p* = 0.131, *p*_adj_ = 0.802 | *p* = 1.000, *p*_adj_ = 1.000 | *p* = 0.343, *p*_adj_ = 1.000 |
| Rater 3 | *p* = 1.000, *p*_adj_ = 1.000 | *p* = 0.6171, *p*_adj_ = 1.000 | *p* = 1.000, *p*_adj_ = 1.000 | *p* = 1.000, *p*_adj_ = 1.000 |

**Supplementary Table 2:** Pairwise comparisons of diagnostic accuracy using McNemar’s test. The table displays the results of pairwise McNemar tests comparing the diagnostic accuracy (proportion of correct classifications) of each novel imaging scenario (CTh map, z-score maps, 3D T1 + CTh map, 3D T1 + z-score map) against the conventional 3D T1 reading condition. Results are stratified by the three independent raters. For each comparison, *p*-value as well as the adjusted *p* -value (*p*_adj_) using *Benjamini-Hochberg* correction are reported. Statistical significance is defined as *p* < 0.05.

| Scenario | | 3D T1 | | | | CTh map | | | | z-score map | | | | CTh map + 3D T1 | | | | z-score map + 3D T1 | | | |
| --- | --- | --- | --- | --- | --- | --- | --- | --- | --- | --- | --- | --- | --- | --- | --- | --- | --- | --- | --- | --- | --- |
| A) Rater 1 | | **Predicted** | | | | | | | | | | | | | | | | | | | |
|  |  | **AD** | **PCA** | **bvFTD** | **svPPA** | **AD** | **PCA** | **bvFTD** | **svPPA** | **AD** | **PCA** | **bvFTD** | **svPPA** | **AD** | **PCA** | **bvFTD** | **svPPA** | **AD** | **PCA** | **bvFTD** | **svPPA** |
| Actual | **AD** | 7 | 0 | 0 | 1 | 3 | 4 | 1 | 0 | 5 | 1 | 1 | 0 | 4 | 3 | 2 | 0 | 5 | 3 | 1 | 1 |
|  | **PCA** | 3 | 5 | 1 | 0 | 1 | 8 | 0 | 0 | 1 | 4 | 0 | 0 | 3 | 7 | 0 | 0 | 2 | 6 | 0 | 2 |
|  | **bvFTD** | 2 | 0 | 5 | 1 | 2 | 0 | 6 | 0 | 2 | 0 | 6 | 1 | 2 | 0 | 7 | 0 | 3 | 0 | 6 | 0 |
|  | **svPPA** | 1 | 0 | 0 | 9 | 2 | 0 | 1 | 5 | 0 | 0 | 1 | 9 | 1 | 0 | 1 | 8 | 0 | 0 | 2 | 8 |
| B) Rater 2 | | **Predicted** | | | | | | | | | | | | | | | | | | | |
|  |  | **AD** | **PCA** | **bvFTD** | **svPPA** | **AD** | **PCA** | **bvFTD** | **svPPA** | **AD** | **PCA** | **bvFTD** | **svPPA** | **AD** | **PCA** | **bvFTD** | **svPPA** | **AD** | **PCA** | **bvFTD** | **svPPA** |
| Actual | **AD** | 2 | 4 | 0 | 3 | 3 | 4 | 0 | 2 | 4 | 2 | 1 | 1 | 4 | 3 | 1 | 0 | 4 | 2 | 0 | 2 |
|  | **PCA** | 4 | 6 | 0 | 0 | 3 | 7 | 0 | 0 | 6 | 3 | 0 | 1 | 5 | 5 | 0 | 0 | 2 | 5 | 0 | 2 |
|  | **bvFTD** | 2 | 0 | 5 | 1 | 1 | 0 | 7 | 0 | 0 | 0 | 8 | 1 | 0 | 0 | 7 | 1 | 0 | 0 | 7 | 2 |
|  | **svPPA** | 4 | 0 | 0 | 5 | 0 | 0 | 0 | 9 | 0 | 0 | 1 | 9 | 1 | 0 | 0 | 9 | 0 | 0 | 1 | 9 |
| B) Rater 3 | | **Predicted** | | | | | | | | | | | | | | | | | | | |
|  |  | **AD** | **PCA** | **bvFTD** | **svPPA** | **AD** | **PCA** | **bvFTD** | **svPPA** | **AD** | **PCA** | **bvFTD** | **svPPA** | **AD** | **PCA** | **bvFTD** | **svPPA** | **AD** | **PCA** | **bvFTD** | **svPPA** |
| Actual | **AD** | 6 | 1 | 1 | 1 | 6 | 2 | 2 | 0 | 7 | 2 | 1 | 0 | 3 | 5 | 2 | 0 | 6 | 1 | 1 | 0 |
|  | **PCA** | 1 | 6 | 0 | 2 | 1 | 8 | 0 | 0 | 3 | 6 | 0 | 0 | 2 | 8 | 0 | 0 | 3 | 5 | 0 | 0 |
|  | **bvFTD** | 1 | 0 | 7 | 2 | 1 | 0 | 6 | 2 | 0 | 0 | 9 | 1 | 1 | 0 | 6 | 2 | 0 | 0 | 9 | 1 |
|  | **svPPA** | 6 | 0 | 0 | 4 | 2 | 0 | 1 | 7 | 0 | 0 | 1 | 9 | 6 | 0 | 0 | 4 | 1 | 0 | 0 | 9 |

**Supplementary Table 3:** Confusion matrix for differentiation of neurodegenerative disease entities for (**A**) Rater 1, (**B**) Rater 2, and **(C)** Rater 3 for the five different rating scenarios.

| AD | 3D T1 | CTh map | z-score map | CTh map + 3D T1 | z-score map + 3D T1 |
| --- | --- | --- | --- | --- | --- |
| A) Rater 1 | | | | | |
| ACC | **0.800** (0.641 - 0.900) | **0.697** (0.527 - 0.826) | **0.839** (0.674 - 0.929) | **0.711** (0.552 - 0.830) | **0.744** (0.589 - 0.854) |
| Sens | **0.875** (0.529 - 0.978) | **0.375** (0.137 - 0.694) | **0.714** (0.359 - 0.918) | **0.444** (0.189 - 0.733) | **0.500** (0.237 - 0.763) |
| Spec | **0.778** (0.592 - 0.894) | **0.800** (0.609 - 0.911) | **0.875** (0.690 - 0.957) | **0.793** (0.616 - 0.902) | **0.828** (0.655 - 0.924) |
| PPV | **0.538** (0.291 - 0.768) | **0.375** (0.137 - 0.694) | **0.625** (0.306 - 0.863) | **0.400** (0.168 - 0.687) | **0.500** (0.237 - 0.763) |
| NPV | **0.955** (0.782 - 0.992) | **0.800** (0.609 - 0.911) | **0.913** (0.732 - 0.976) | **0.821** (0.644 - 0.921) | **0.828** (0.655 - 0.924) |
| B) Rater 2 | | | | | |
| ACC | **0.528** (0.370 - 0.680) | **0.722** (0.560 - 0.842) | **0.730** (0.570 - 0.846) | **0.722** (0.560- 0.842) | **0.833** (0.681 - 0.921) |
| Sens | **0.222** (0.063 - 0.547) | **0.333** (0.121 - 0.646) | **0.500** (0.215 - 0.785) | **0.500** (0.215 - 0.785) | **0.500** (0.215 - 0.785) |
| Spec | **0.630** (0.442 - 0.785) | **0.852** (0.675 - 0.941) | **0.793** (0.616 - 0.902) | **0.786** (0.605 - 0.898) | **0.929** (0.774 - 0.980) |
| PPV | **0.167** (0.047 - 0.448) | **0.429** (0.158 - 0.750) | **0.400** (0.168 - 0.687) | **0.400** (0.168 - 0.687) | **0.667** (0.300 - 0.903) |
| NPV | **0.708** (0.508 - 0.851) | **0.793** (0.616 - 0.902) | **0.852** (0.675 - 0.941) | **0.846** (0.665 - 0.938) | 0.867 (0.703 - 0.947) |
| C) Rater 3 | | | | | |
| ACC | **0.711** (0.552, 0.830) | **0.789** (0.637, 0.889) | **0.846** (0.703, 0.928) | **0.590** (0.434, 0.729) | **0.833** (0.681, 0.921) |
| Sens | **0.667** (0.354, 0.879) | **0.600** (0.313, 0.832) | **0.700** (0.397, 0.892) | **0.300** (0.108, 0.603) | **0.750** (0.409, 0.929) |
| Spec | **0.724** (0.543, 0.853) | **0.857** (0.685, 0.943) | **0.897** (0.736, 0.964) | **0.690** (0.508, 0.827) | **0.857** (0.685, 0.943) |
| PPV | **0.429** (0.214, 0.674) | **0.600** (0.313, 0.832) | **0.700** (0.397, 0.892) | **0.250** (0.089, 0.532) | **0.600** (0.313, 0.832) |
| NPV | **0.875** (0.690, 0.957) | **0.857** (0.685, 0.943) | **0.897** (0.736, 0.964) | **0.741** (0.553, 0.868) | **0.923** (0.759, 0.979) |

**Supplementary Table 4:** One vs. all performance metrics for AD (means of point estimates with 95 % confidence intervals calculated using the Wilson method) for **(A)** Rater 1, **(B)** Rater 2, and **(C)** Rater 3 for the five different scenarios.

| PCA | 3D T1 | CTh map | z-score map | CTh map + 3D T1 | z-score map + 3D T1 |
| --- | --- | --- | --- | --- | --- |
| A) Rater 1 | | | | | |
| ACC | **0.886** (0.740 - 0.955) | **0.848** (0.691 - 0.933) | **0.935** (0.792 - 0.982) | **0.842** (0.696 - 0.926) | **0.821** (0.673 - 0.910) |
| Sens | **0.556** (0.267 - 0.811) | **0.889** (0.565 - 0.980) | **0.800** (0.376 - 0.964) | **0.700** (0.397 - 0.892) | **0.600** (0.313 – 0.832) |
| Spec | **1.000** (0.871 - 1.000) | **0.833** (0.641 - 0.933) | **0.962** (0.811 - 0.993) | **0.893** (0.728 - 0.963) | **0.897** (0.736 - 0.964) |
| PPV | **1.000** (0.566 - 1.000) | **0.667** (0.391 - 0.862) | **0.800** (0.376 - 0.964) | **0.700** (0.397 - 0.892) | **0.667** (0.354 - 0.879) |
| NPV | **0.867** (0.703 - 0.947) | **0.952** (0.773 - 0.992) | **0.962** (0.811 - 0.993) | **0.893** (0.728 - 0.963) | **0.867** (0.703 - 0.947) |
| B) Rater 2 | | | | | |
| ACC | **0.778** (0.619 - 0.883) | **0.806** (0.650 - 0.902) | **0.757** (0.599 - 0.866) | **0.778** (0.619 - 0.883) | **0.833** (0.681 - 0.921) |
| Sens | **0.600** (0.313 - 0.832) | **0.700** (0.397 - 0.892) | **0.300** (0.108 - 0.603) | **0.500** (0.237 - 0.763) | **0.556** (0.267 - 0.811) |
| Spec | **0.846** (0.665 - 0.938) | **0.846** (0.665 - 0.938) | **0.926** (0.766 - 0.979) | **0.885** (0.710 - 0.960) | **0.926** (0.766 - 0.979) |
| PPV | **0.600** (0.313- 0.832) | **0.636** (0.354 - 0.948) | **0.600** (0.231 - 0.882) | **0.625** (0.306 - 0.863) | **0.714** (0.359 - 0.918) |
| NPV | **0.846** (0.665 - 0.938) | **0.880** (0.700 - 0.958) | **0.781** (0.612 - 0.890) | **0.821** (0.644 - 0.921) | **0.862** (0.694 - 0.945) |
| C) Rater 3 | | | | | |
| ACC | **0.895** (0.759, 0.958) | **0.921** (0.792, 0.973) | **0.872** (0.733, 0.944) | **0.821** (0.673, 0.910) | **0.889** (0.747, 0.956) |
| Sens | **0.667** (0.354, 0.879) | **0.889** (0.565, 0.980) | **0.667** (0.354, 0.879) | **0.800** (0.490, 0.943) | **0.625** (0.306, 0.863) |
| Spec | **0.966** (0.828, 0.994) | **0.931** (0.780, 0.981) | **0.933** (0.787, 0.982) | **0.828** (0.655, 0.924) | **0.964** (0.823, 0.994) |
| PPV | **0.857** (0.487, 0.974) | **0.800** (0.490, 0.943) | **0.750** (0.409, 0.929) | **0.615** (0.355, 0.823) | **0.833** (0.436, 0.970) |
| NPV | **0.903** (0.751, 0.967) | **0.964** (0.823, 0.994) | **0.903** (0.751, 0.967) | **0.923** (0.759, 0.979) | **0.900** (0.744, 0.965) |

**Supplementary Table 5:** One vs. all performance metrics for PCA (means of point estimates with 95 % confidence intervals calculated using the Wilson method) for **(A)** Rater 1**, (B)** Rater 2, and **(C)** Rater 3 for the five different scenarios.

| bvFTD | 3D T1 | CTh map | z-score map | CTh map + 3D T1 | z-score map + 3D T1 |
| --- | --- | --- | --- | --- | --- |
| A) Rater 1 | | | | | |
| ACC | **0.886** (0.740 - 0.955) | **0.879** (0.727 - 0.952) | **0.839** (0.674 – 0.929) | **0.868** (0.727 - 0.942) | **0.846** (0.703 - 0.928) |
| Sens | **0.625** (0.306 - 0.863) | **0.750** (0.409 - 0.929) | **0.667** (0.354 - 0.879) | **0.778** (0.453 - 0.937) | **0.667** (0.354 - 0.879) |
| Spec | **0.963** (0.817 - 0.993) | **0.920** (0.750 - 0.978) | **0.909** (0.722 - 0.975) | **0.897** (0.736 - 0.964) | **0.900** (0.744 - 0.965) |
| PPV | **0.833** (0.436 - 0.970) | **0.750** (0.409 - 0.929) | **0.750** (0.409 - 0.929) | **0.700** (0.397 - 0.892) | **0.667** (0.354 - 0.879) |
| NPV | **0.897** (0.736 - 0.964) | **0.920** (0.750 - 0.978) | **0.870** (0.679 - 0.955) | **0.929** (0.774 - 0.980) | **0.900** (0.744 - 0.965) |
| B) Rater 2 | | | | | |
| ACC | **0.917** (0.782 – 0.971) | **0.972** (0.858 – 0.995) | **0.919** (0.787 – 0.972) | **0.944** (0.819 - 0.985) | **0.917** (0.513 - 1.000) |
| Sens | **0.625** (0.306 - 0.863) | **0.875** (0.529 - 0.978) | **0.889** (0.565 - 0.980) | **0.875** (0.529 - 0.978) | **0.778** (0.453 - 0.937) |
| Spec | **1.000** (0.879 - 1.000) | **1.000** (0.879 - 1.000) | **0.929** (0.774 - 0.980) | **0.964** (0.823 - 0.994) | **0.963** (0.817 - 0.993) |
| PPV | **1.000** (0.566 - 1.000) | **1.000** (0.646 - 1.000) | **0.800** (0.490 - 0.943) | **0.875** (0.529 - 0.978) | **0.875** (0.529 - 0.978) |
| NPV | **0.903** (0.751 - 0.967) | **0.966** (0.828 - 0.994) | **0.963** (0.817 - 0.993) | **0.964** (0.823 - 0.994) | **0.929** (0.774 - 0.980) |
| C) Rater 3 | | | | | |
| ACC | **0.895** (0.759, 0.958) | **0.842** (0.696, 0.926) | **0.923** (0.797, 0.973) | **0.872** (0.733, 0.944) | **0.944** (0.819, 0.985) |
| Sens | **0.700** (0.397, 0.892) | **0.667** (0.354, 0.879) | **0.900** (0.596, 0.982) | **0.667** (0.354, 0.879) | **0.900** (0.596, 0.982) |
| Spec | **0.964** (0.823, 0.994) | **0.897** (0.736, 0.964) | **0.931** (0.780, 0.981) | **0.933** (0.787, 0.982) | **0.962** (0.811, 0.993) |
| PPV | **0.875** (0.529, 0.978) | **0.667** (0.354, 0.879) | **0.818** (0.523, 0.949) | **0.750** (0.409, 0.929) | **0.900** (0.596, 0.982) |
| NPV | **0.900** (0.744, 0.965) | **0.897** (0.736, 0.964) | **0.964** (0.823, 0.994) | **0.903** (0.751, 0.967) | **0.962** (0.811, 0.993) |

**Supplementary Table 6:** One vs. all performance metrics for bvFTD (means of point estimates with 95 % confidence intervals calculated using the Wilson method) for **(A)** Rater 1, **(B)** Rater 2, and **(C)** Rater 3 for the five different scenarios.

| svPPA | 3D T1 | CTh map | z-score map | CTh map + 3D T1 | z-score map + 3D T1 |
| --- | --- | --- | --- | --- | --- |
| A) Rater 1 | | | | | |
| ACC | **0.914** (0.776 - 0.970) | **0.909** (0.764 - 0.969) | **0.935** (0.793 - 0.982) | **0.950** (0.835 - 0.986) | **0.872** (0.733 - 0.944) |
| Sens | **0.900** (0.596 - 0.982) | **0.625** (0.306 - 0.863) | **0.900** (0.596 - 0.982) | **0.800** (0.490 - 0.943) | **0.800** (0.490 - 0.943) |
| Spec | **0.920** (0.750 - 0.978) | **1.000** (0.867 - 1.000) | **0.952** (0.773 - 0.992) | **1.000** (0.879 - 1.000) | **0.897** (0.736 - 0.964) |
| PPV | **0.818** (0.523 - 0.949) | **1.000** (0.566 - 1.000) | **0.900** (0.596 - 0.982) | **1.000** (0.676 - 1.000) | **0.727** (0.434 - 0.903) |
| NPV | **0.958** (0.798 - 0.993) | **0.893** (0.728 - 0.963) | **0.952** (0.773 - 0.992) | **0.933** (0.787 - 0.982) | **0.929** (0.774 - 0.980) |
| B) Rater 2 | | | | | |
| ACC | **0.778** (0.619 - 0.883) | **0.944** (0.819 - 0.985) | **0.892** (0.753 - 0.957) | **0.944** (0.819 - 0.985) | **0.806** (0.650 - 0.902) |
| Sens | **0.556** (0.267 - 0.811) | **1.000** (0.766 - 1.000) | **0.900** (0.596 - 0.982) | **0.900** (0.596 - 0.982) | **0.900** (0.596 - 0.982) |
| Spec | **0.852** (0.675 - 0.941) | **0.926** (0.780 - 0.981) | **0.889** (0.719 - 0.961) | **0.962** (0.811 - 0.993) | **0.769** (0.579 - 0.890) |
| PPV | **0.556** (0.267 - 0.811) | **0.818** (0.523 - 0.949) | **0.750** (0.468 - 0.911) | **0.900** (0.596 - 0.982) | **0.600** (0.357 - 0.802) |
| NPV | **0.852** (0.675 - 0.941) | **1.000** (0.867 - 1.000) | **0.960** (0.811 - 0.993) | **0.962** (0.828 - 0.994) | **0.952** (0.773 - 0.992) |
| C) Rater 3 | | | | | |
| ACC | **0.711** (0.552, 0.830) | **0.868** (0.727, 0.942) | **0.949** (0.831, 0.986) | **0.795** (0.645, 0.892) | **0.944** (0.819, 0.985) |
| Sens | **0.400** (0.168, 0.687) | **0.700** (0.397, 0.892) | **0.900** (0.596, 0.982) | **0.400** (0.168, 0.687) | **0.900** (0.596, 0.982) |
| Spec | **0.821** (0.644, 0.921) | **0.929** (0.774, 0.980) | **0.966** (0.828, 0.994) | **0.931** (0.780, 0.981) | **0.962** (0.811, 0.993) |
| PPV | **0.444** (0.189, 0.733) | **0.778** (0.453, 0.937) | **0.900** (0.596, 0.982) | **0.667** (0.300, 0.903) | **0.900** (0.596, 0.982) |
| NPV | **0.793** (0.616, 0.902) | **0.897** (0.736, 0.964) | **0.966** (0.828, 0.994) | **0.818** (0.656, 0.914) | **0.962** (0.811, 0.993) |

**Supplementary Table 7:** One vs. all performance metrics for svPPA (means of point estimates with 95 % confidence intervals calculated using the Wilson method) for **(A)** Rater 1, (**B)** Rater 2, and **(C)** Rater 3 for the five different scenarios.

|  | 3D T1 vs CTh | 3D T1 vs z-score | 3D T1 vs CTh map + 3D T1 | 3D T1 vs z-score map + 3D T1 |
| --- | --- | --- | --- | --- |
| Rater 1 | *p* = 0.024, *p*_adj_ = 0.097 | *p* = 0.673*, p*_adj_ = 0.735 | *p* = 0.742*, p*_adj_ = 0.742 | *p* = 0.183, *p*_adj_ = 0.307 |
| Rater 2 | *p* = 0.184, *p*_adj_ = 0.307 | *p* =0.003, *p*_adj_ = 0.017 | *p* = 0.038, *p*_adj_ = 0.115 | *p* = 0.003, *p*_adj_ = 0.017 |
| Rater 3 | *p* = 0.433, *p*_adj_ = 0.577 | *p* =0.181, *p*_adj_ = 0.307 | *p* = 0.586*, p*_adj_ = 0.703 | *p* = 0.205, *p*_adj_ = 0.307 |

**Supplementary Table 8:** Pairwise comparisons of diagnostic confidence ratings for detection of any neurodegenerative disease versus healthy controls. The table shows the results of two-sided Wilcoxon signed-rank tests comparing raters' ordinal confidence scores (1=low, 2=medium, 3=high) between the conventional 3D T1 reading condition and the novel imaging scenarios (CTh and z-score maps alone or combined with 3D T1).  *p*-values were adjusted for multiple comparisons within each rater using the *Benjamini-Hochberg* procedure. Statistical significance was set at an adjusted *p* < 0.05.

|  | 3D T1 vs CTh | 3D T1 vs z-score | 3D T1 vs CTh map + 3D T1 | 3D T1 vs z-score map + 3D T1 |
| --- | --- | --- | --- | --- |
| Rater 1 | *p* = 0.269, *p*_adj_ = 0.414 | *p* = 0.736*, p*_adj_ = 0.802 | *p* =0.204*, p*_adj_ = 0.414 | *p* = 0.133, *p*_adj_ = 0.399 |
| Rater 2 | *p* = 0.883, *p*_adj_ = 0.883 | *p* =0.104, *p*_adj_ = 0.399 | *p* = 0.580, *p*_adj_ = 0.696 | *p* = 0.038, *p*_adj_ = 0.227 |
| Rater 3 | *p* = 0.313, *p*_adj_ = 0.418 | *p* =0.224, *p*_adj_ = 0.414 | *p* = 0.277*, p*_adj_ = 0.414 | *p* = 0.025, *p*_adj_ = 0.227 |

**Supplementary Table 9:** Pairwise comparisons of diagnostic confidence ratings for multiclass differentiation of neurodegenrative disease entities. The table shows the results of two-sided Wilcoxon signed-rank tests comparing raters' ordinal confidence scores (1=low, 2=medium, 3=high) between the conventional 3D T1 reading condition and the novel imaging scenarios (CTh and z-score maps alone or combined with 3D T1).  *p*-values were adjusted for multiple comparisons within each rater using the *Benjamini-Hochberg* procedure. Statistical significance was set at an adjusted *p* < 0.05.

| Scenario | 3D T1 | CTh map | z-score map | CTh map + 3D T1 | z-score map + 3D T1 |
| --- | --- | --- | --- | --- | --- |
| Per-class κ for AD | **0.200** (-0.000, 0.400) | **0.496** (0.293, 0.699) | **0.408** (0.201, 0.614) | **0.169** (-0.019, 0.358) | **0.189** (-0.005, 0.383) |
| Per-class κ for PCA | **0.571** (0.371, 0.771) | **0.859** (0.656, 1.062) | **0.710** (0.503, 0.917) | **0.593** (0.404, 0.781) | **0.528** (0.334, 0.722) |
| Per-class κ for bvFTD | **0.428** (0.228, 0.628) | **0.762** (0.559, 0.965) | **0.896** (0.690, 1.103) | **0.558** (0.369, 0.747) | **0.587** (0.393, 0.781) |
| Per-class κ for svPPA | **0.209** (0.009, 0.409) | **0.631** (0.428, 0.834) | **0.951** (0.744, 1.157) | **0.571** (0.383, 0.760) | **0.571** (0.377, 0.765) |

**Supplementary Table 10:** Fleiss’s κ with 95 % confidence intervals calculated using the asymptotic standard error method for interrater agreement for differentiation of neurodegenerative disease entity per class (AD, PCA, bvFTD, and svPPA).
